# New Quartz Oscillator Switching Method for Nano-Henry Range Inductance Measurements

**DOI:** 10.3390/s120303105

**Published:** 2012-03-06

**Authors:** Vojko Matko, Karel Jezernik

**Affiliations:** Faculty of Electrical Engineering and Computer Science, University of Maribor, Smetanova 17, 2000 Maribor, Slovenia; E-Mail: karel.jezernik@uni-mb.si

**Keywords:** nano-Henry range measurement of small inductance changes, switching oscillating method, compensation of quartz crystal temperature characteristics

## Abstract

This article introduces a new method for nano-Henry inductance measurements at the frequency of 4.999 MHz with a single quartz crystal oscillating in the switching oscillating circuit. The real novelty of this method, however, lies in a considerable reduction of the temperature influence of AT-cut crystal frequency change in the temperature range between 0 °C and 50 °C through a switching method which compensates for the crystal’s natural temperature characteristics. This allows for the compensation of any influences on the crystal such as the compensation of the non-linear temperature characteristics and the ageing of both the crystal and other oscillating circuit elements, as well as the reduction of the output frequency measurement errors with the help of an additional reference frequency. The experimental results show that the switching method greatly improves the measurement of small inductance changes in the range between μH and nH, allowing as a result high-precision measurements (∼0.35 fH) in this range.

## 1. Introduction

When using existing bridge methods for inductance measurement, one experiences the problem of the occurrence of stray capacitances and inductances once all bridge elements are connected. Also, the range of measurement is generally limited to just 100 kHz. In the Maxwell-Wien bridge, the measurement range is between 20 Hz and 1 MHz and the relative error of measurement is about 0.1% of the measured values. The resonance methods, on the other hand, are based on the application of a series or parallel resonance LC circuits as elements of either a bridge circuit or a two-port (four-terminal) “T”-type network. A “double T” network can be used for inductive impedance measurements at high frequencies (up to 100 MHz; the relative error of measurement is about 0.1%). Proper design of LCR meters limits the influence of the factors causing error of measurement (*i.e*., stray coupling and distortions). The results of inductance measurements also depend on how the measurements are performed, including how the measured inductor is connected to the meter. Care should be taken to limit such influences as inductive and capacitive coupling of the inductive element to the environment, sources of distortion, and the choice of operating frequency. One such precise LCR meter is also 4284A Hewlett-Packard meter, whose range of measurement is between 0.01 nH and 99.9999 kH in the frequency range 20 Hz–1 MHz with the basic accuracy of 0.05%. However, its price is relatively expensive ∼$9,500 [1–6].

To improve the precision and sensitivity of small inductance change measurements using quartz crystals, a switching configuration has been made. Approaches described in articles to date [7–10] achieved greater precision through compensation of various influences from non-linear temperature characteristics to ageing and frequency measurement influences (errors). Sensitivity, on the other hand, was increased using various techniques such as frequency pulling with the prime aim of achieving high sensitivity in the nano-Henry range. However, the inequality of quartz crystal pair temperature characteristics has limited research in that direction [1].

Differential quartz crystal oscillating measurement methods typically consist of two oscillators using two quartz crystals (temperature pairs) [1,2,10,11], whereby these temperature pairs usually do not have ideal temperature characteristics. This means that the temperature compensation influence can be achieved only to a certain degree and only within a limited temperature range. The results of past experiments show that quartz crystal temperature characteristics are of primary importance in high-precision measurement of small inductances. Equally important are a stable electronic oscillating circuit supply voltage and the related quality of individual elements [1–6].

In cases where the oscillator housing is not hermetically closed, one should also take into account the influence of the outside environment on the outside oscillator elements. The key thing is to have these elements made of high-quality stable materials such as Al_2_O_3_, which is known for its very good temperature stability (coefficient of thermal expansion—8.1 × 10^−6^/°C, dielectric constant—9.1 (1 MHz), maximum working temperature—1,700 °C, hardness—1,175 Kg/mm^2^, tensile strength—107 MPa, compressive strength—1,288 MPa, bending strength—248 MPa, porosity—0%,…) [10–13].

The research described in this article introduces a new idea which looks for ways to compensate the temperature characteristics of a single quartz crystal. The real novelty of this approach lies in the fact that AT-cut quartz crystals with various temperature characteristics can be used. Similarly, the aim is also to compensate the temperature influence of the measuring inductances on the quartz crystal oscillation frequency, the influence of the supply voltage on the oscillating circuit as well as the influence of any other electronic circuit element, and to foresee the differential functioning of the sensitive inductive elements [10,14,15].

Switching circuits may sometimes have some disadvantages, which can, however, be greatly minimized using appropriate approaches. In case of oscillator circuits, an oscillator with a good start-up, *i.e*., with a reliable crystal oscillation during the start and later on, is a must. We refer to this as short- and long-term frequency temperature stability. Dynamic stability during the temperature changes in the extended operating range is another factor of great importance [14–20].

Typical frequency measurement is limited to the counter capacities, *i.e*., to the errors in a given frequency range. At higher frequency measurements, the heterodyne measurement method proves to be better. In this method, the frequency difference is transformed to a lower range, where it can be measured with a substantially higher precision than the original high frequency. For such measurements, instruments such as counter (HM 8122) are typically used [1,15,18].

There is also the problem of how to combine and implement high sensitivity (frequency pulling), linearize frequency inductance characteristics, set the crystal oscillation frequency working point, compensate other influences and ensure high temperature stability discussed by previous articles. The best frequency range to achieve high pulling sensitivity and stability for high quality crystals is between 3 and 10 MH. The problem of load pulling becomes worse at higher frequencies, because both the quality (Q) and isolation of the quartz crystal decrease. The frequency stability also decreases due to lower Q value [1,8,9,19–25].

## 2. Dual Switching Mode Oscillator

**Figure 1.**
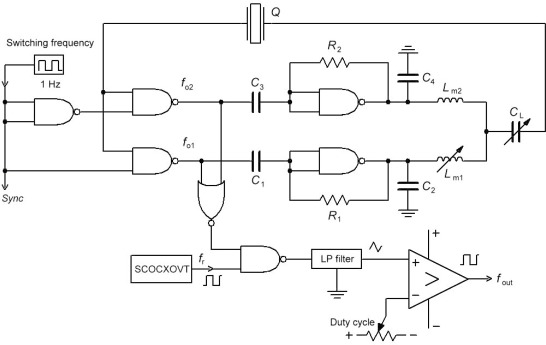
Dual switching mode oscillator.

A dual switching mode oscillator is based on one quartz crystal and a dual oscillator circuit with switching part together (Figure 1) [1,14,19]. The switching between the frequencies *f*_o1_ and *f*_o2_ is done by an additional switching frequency (*Sync*) of 1 Hz and an additional circuit of three NAND gates. Inductances *L*_m1_ and *L*_m2_ can also be used as sensors in differential mode. Inductors can be produced in various standard forms. They can generally be classified as strip inductors or spiral inductors. Straight sections of wires or strip are used for low inductance values, typically less than 10 nH, while spiral have higher quality Q_s_ and can provide higher inductance values. The presence of a ground plane also affects the inductance. The inductance decreases when the ground plane is brought nearer to the conducting line. Planar inductors are made essentially with a single-layer metallization scheme, in which a conducting layer is etched on a dielectric substrate. In the experiment, *L*_m1_ consists of two inductances in the series—standard element (123 μH) and straight sections of strip for low inductance values (2 nH) (etched on Al_2_O_3_). The inductance of a strip line can be written as [26–31]:

(1) $$L_{\mathrm{straight}}=2l\left[\text{ln}\left(\frac{l}{w+t}\right)+0.22\frac{w+t}{l}+1.19\right]$$

Where *L_straight_* is the segment inductance in nH, *l* = 0.5 cm, *w* = 0.05 cm, and *t* = 0.05 cm are the segment length, width and thickness, respectively. By changing the length *l* (using constant values), inductance values of *L_straight_* in the range between 0 and 2 nH can be set. Inductance *L_straight_* was measured by a HP 4194A impedance/gain-phase analyzer. Inductance *L*_m2_ has the value of 123 μH. The basic values of inductances *L*_m1_ and *L*_m2_ are determined with the compensation criterion (Equation (2)) in relation to the parasitic capacitance *C*_o_ = 8 pF of the quartz crystal [1,8,19,26]:

(2) $$k\cdot L_{m}=1/k\cdot C_{0}$$

The two inductances *L*_m1_ and *L*_m2_ play the roles of sensor and compensation elements at the same time, which is another of the novelties introduced by this method. Capacitance *C*_L_ is used for the simultaneous fine tuning of the frequencies *f*_o1_ and *f*_o2_. The compensation factor *k* can be set with the fixed values *L*_m1_ and *L*_m2_ [7,8,26,27].

The signal corresponding to the difference between the frequencies enters the LP filter. At the LP filter output, the triangular signal is produced which is then converted with the help of a comparator to a rectangular signal representing the output signal. The output *f*_out_ thus represents the output frequency signal which is synchronously measured with regard to the switch of signal *Sync*. Capacitances *C*_2_ and *C*_4_ serve to suppress the spurious responses to avoid crystal oscillation at higher and/or lower frequencies [19,20,32]. Depending upon the sensor design and configuration, the spurious response modes should be weaker than the fundamental mode. The only thing is that they are taking away the energy from the mode of interest. In the oscillator circuit, the inductances *L*_m1_ and *L*_m2_ are in series with the quartz crystal and together with the compensation method (*C*_0_) increase and linearize the frequency pulling range [8,9]. When inductances *L*_m1_ and *L*_m2_ are the same, *f*_out_ remains the same at state 1 and 0 of *Sync* signal and depends on the quartz crystal resonant frequency *f*_0_, quartz crystal temperature characteristics Δ*f*(*T*), its ageing Δ*f*(*t*) and the Δ*C_L_* change. However, when the inductances *L*_m1_ and *L*_m2_ are different, the frequency *f*_out_ depends on the quartz crystal resonant frequency *f*_0_, quartz crystal temperature characteristics Δ*f*(*T*), its ageing Δ*f*(*t*), and the Δ*C_L_* or Δ*L_m_*_1_ change. In case of the difference of the two frequencies *f*_o1_ in *f*_o2_, Δ*f*(*T*), Δ*C_L_* and Δ*f*(*t*) are compensated because only one quartz characteristics is involved [14,19,20].

The output frequencies *f*_out_ depend on *Sync* signal and can be expanded to [14]:

(3) $$f(\mathrm{Sync})-f_{r}=f_{0}+\Delta f(T)+\Delta f(t)+\Delta C_{L}+\Delta f(\mathrm{counter}\;\mathrm{error})+\Delta f(L_{m1})+\Delta f(\Delta L_{m1})-f_{r}$$

(4) $$f(\bar{\mathrm{Sync}})-f_{r}=f_{0}+\Delta f(T)+\Delta f(t)+\Delta C_{L}+\Delta f(\mathrm{counter}\;\mathrm{error})+\Delta f(L_{m2})-f_{r}$$

when joining *f*_0_ and Δ*f*(*L*_*m*1_ + Δ*L*_*m*1_), we get Equation (5). The particularity of this equation lies in the fact that it takes into account the compensation C_0_ and at the same time linearizes the quartz characteristics due to the Δ*L*_*m*1_ change (Figure 1) and allows for the sensitivity setting [14,19,20].

(5) $$f(\mathrm{Sync},\;k,\;\Delta L_{m1})=\frac{1+\frac{C}{2\left(\frac{1}{k}\;C_{0}-\frac{1}{\omega_{0}{}^{2}\cdot k\cdot (L_{m1}+\Delta L_{m1})-\frac{1}{C_{L}}}\right)}}{2\pi \cdot \sqrt{L\cdot C}}+\Delta f(T)+\Delta f(t)+\Delta f(\mathrm{counter}\;\mathrm{error})$$

where:

*k*_1_, *k*_2_, *k*_3_—pulling sensitivity value [8],
*L* and *C*—mechanical behavior of the crystal element [8,33–36],
*L*_m1_—measurement and compensation inductance 1 [33–36],
*L*_m2_—compensation inductance 2,
*C*_0_—parasitic capacitance of the crystal element and holder [8,33–36],
*f*_0_—quartz crystal series resonant frequency [8,33–36],
*T*—temperature,
*t*—time.

(6) $$\omega_{0}=2\cdot \pi \cdot f_{0}$$

when joining *f*_0_ and Δ*f*(*L*_*m*2_), we get Equation (7) [10,14]:

(7) $$f(\bar{\mathrm{Sync}},\;k,\;L_{m2})=\frac{1+\frac{C}{2\left(\frac{1}{k}\;C_{0}-\frac{1}{\omega_{0}{}^{2}\cdot k\cdot (L_{m2})-\frac{1}{C_{L}}}\right)}}{2\pi \cdot \sqrt{L\cdot C}}+\Delta f(T)+\Delta f(t)+\Delta f(\mathrm{counter}\;\mathrm{error})$$

The pulling sensitivity in Equations (5) and (7) can be set with the value *k*, achieving at the same time simultaneous dependence linearization Δ*f*(*L_m_*_1_ + Δ*L_m_*_1_) [7,8]. At every switch between *Sync* and $\bar{\mathrm{Sync}}$, the frequency *f*_out_ is measured by counter and its value is transferred to the LabVIEW software calculating the difference between the two frequencies. The switching from *Sync* to $\bar{\mathrm{Sync}}$ also compensates the auxiliary frequency *f*_r_, and consequently its frequency temperature stability as well. We get the frequency difference representing the temperature compensated and linear value of the frequency, which depends uniquely on the Δ*L*_*m*1_ change:

(8) $$\left(f(\mathrm{Sync},\;k,\;L_{m1}+\Delta L_{m1})-f_{r})-(f(\bar{\mathrm{Sync}},\;k,\;L_{m2})-f_{r})=\Delta f(\Delta L_{m1}\right)$$

This means that it is neither dependent on the quartz crystal temperature characteristics Δ*f*(*T*) nor its ageing Δ*f*(*t*) and nor the circuit temperature characteristics influences (Equations (8) and (9)) [1,8,14]:

(9) $$\Delta f(\Delta L_{m1})=\frac{\frac{C}{2\left(\frac{1}{k}\;C_{0}-\frac{1}{\omega_{0}{}^{2}\cdot k\cdot (L_{m1}+\Delta L_{m1})-\frac{1}{C_{L}}}\right)}}{2\pi \cdot \sqrt{L\cdot C}}-\frac{1+\frac{C}{2\left(\frac{1}{k}C_{0}-\frac{1}{\omega_{0}{}^{2}\cdot k\cdot (L_{m2})-\frac{1}{C_{L}}}\right)}}{2\pi \cdot \sqrt{L\cdot C}}$$

While the specified counter accuracy (HM 8122—option HO 85) ±5 × 10^−9^ does not allow for high-precision measurements of small output frequency changes at 4.999 MHz, the use of an additional reference frequency *f*_r_ = 4.999 MHz (auxiliary OCOCXOVT oscillator, Figure 1), of the frequency difference method (AND gate) and of the low pass filter enables precise measurements of the frequency difference between the switches *Sync* and $\bar{\mathrm{Sync}}$ [18].

## 3. Quartz Temperature Characteristics Compensation

When using AT-cut crystals for high-precision measurements, a frequency change in the oscillation (up to 1 Hz) of the crystal can be detected in the range between 10 °C and 40 °C [1,14,19–37]. Generally, the different temperature frequency curves are represented as the cubical parabola with temperature inflection point lying between 25 °C and 35 °C, depending on the crystal cut angle and the mechanical construction. The new method (Figure 1) allows the AT-cut crystal temperature characteristics compensation (under 1 Hz) through switching mode method, which compensates this characteristics and reduces its influence to a minimum [14,20,37–39].

In comparison to AT-cut crystals, NLSC-cut crystals are less sensitive to mechanical and thermal stress and provide lower aging and higher Q. Moreover, they are also less sensitive to drive levels. On the other hand, they require an oven, do not operate well at ambient temperatures as the frequency rapidly falls off at lower temperatures. However, since their inflection point is between 85 °C and 95 °C, it is very suitable for ovenized oscillators because a top around 90 °C leads to very low dependency of frequency against temperature [40]. Nevertheless, NLSC-cut crystals have a number of disadvantages, e.g., the motional capacitance is several times less than that of an AT of the same frequency, thus reducing the ability to “pull” the crystal frequency. They are also sensitive to electric fields and their cost is relatively high compared to AT-cut crystals [41–43].

## 4. Frequency Variation of Oscillator as Function of Time

Frequency variation of oscillator as function of time is normally considered in short-term stability (second-to-second temperature characteristics) and long-term stability over years. The short-term stability of a quartz crystal depends on the actual oscillator design and is totally controlled by the quartz crystal at low drive levels. Long-term stability (ageing) is naturally greater during the first part of the life of the crystal unit. The ageing rates of the best cold weld crystals are less than ±1 ppm/year (10 °C to 40 °C) [1,19,32,38,39]. The ageing of other electronic circuit elements is compensated in the same way.

## 5. Output Frequency Measurement Error

Typical counter (HM 8122—option HO 85) accuracy is ±5 × 10^−9^ (through the entire working temperature range 10 °C up to 40 °C), in 5 × 10^−9^/day after 48 h continuous operation with crystal oven controlled (OCXO) [18]. The novel switching method highly reduces the influence of the short- and long-term stability of the above described counter due to the compensation of previously mentioned influences of a single quartz crystal and the circuit as well as the influence of the difference method using additional reference frequency *f*_r_ = 4.999 MHz. Frequency *f*_r_ is produced by oven controlled crystal oscillator (OCOCXOVT) with a short-term stability (0.1 to 30 s) 1 × 10^−10^ in the temperature range between 0 °C and 60 °C following the warm-up time of 30 min [17,18,38,39].

## 6. Experimental Results

For this research, the quartz switching oscillator circuit (Figure 1) was experimentally selected switching between inductances *L*_m1_ and *L*_m2_ with the frequency of 1 Hz. The *L*_m_ values were in the range between 122 μH and 124 μH. Within 1 s time, the HM 8122 external counter measured (using LabVIEW software) both frequencies (*f*(*Sync,k,L*_*m*1_ + Δ*L*_*m*1_) − *f_r_*) and $\left(f(\bar{\mathrm{Sync}},\;k,\;L_{m2})-f_{r}\right)$ at the output *f*_out_.

In Table 1, *f*_0_ represents the fundamental mode quartz crystal resonant frequency. *R*, *L*, *C*, *C*_0_, are quartz crystal equivalent circuit elements (*Q*_q_ is quality) [1,2,7,8,32]. The experimental quartz data values in the quartz crystal equivalent circuit were measured by Jauch [32]. *L*_m1_ and *L*_m2_ were measured using the HP 4194A impedance/gain-phase analyzer.

**Figure 2.**
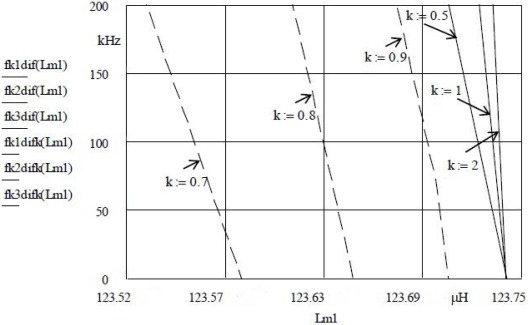
Quartz crystal sensitivity and linearity for *k* = 0.5, 1, 2 for *L*_m1_ + Δ*L*_m1_ and *L*_m2_ = 123.75 μH ·*k*, *C*_L_ = 22 nF.

Figure 2 illustrates the change of inductance values. *L*_m1_ changes for Δ*L*_m1_ with the criterion *k* at the constant capacitance value *C*_L_ = 22 nF. Simultaneously with *L*_m1_, the inductance *L*_m2_ = 123.75 μH is changed with the same criterion *k* (Equation (7)). Figure 2 does not display the inductance *L*_m2_ because in relation to *k* it has a fixed value depending on *k*. Δ*L*_m1_ changes with the change of the length *l* (Equation (1)) [26–31], and is measured by a HP 4194A impedance/gain-phase analyzer. Figure 2 shows that various frequency sensitivities with the intersection at *L*_m1_ = 123.742 μH (due to different *k*) are produced. Factor *k* relates to the compensation criterion *C*_0_ [8]. This means that if we simultaneously change *k (k*_1_ = 0.5, *k*_2_ = 1, *k*_3_ = 2), the size of the frequency sensitivity of *L*_m1_ + Δ *L*_m1_ can be determined with the frequency difference $f(\mathrm{Sync})-f(\bar{\mathrm{Sync})}$ for (*f*_k1dif_(*L*_m1_), *f*_k2dif_(*L*_m1_), *f*_k3dif_(*L*_m1_).

Figure 2 also shows that by changing the inductance values of *L*_m1_ alone with the values of the criterion *k* = 0.7, 0.8, 0.9 at the constant capacitance value *C*_L_ = 22 nF and *k* = 1 for fixed *L*_m2_ = 123.75 H in all three cases, the oscillator frequency can be set to the left and to the right of the initially set frequency by changing the value of *k* for *L*_m1_ alone.

**Figure 3.**
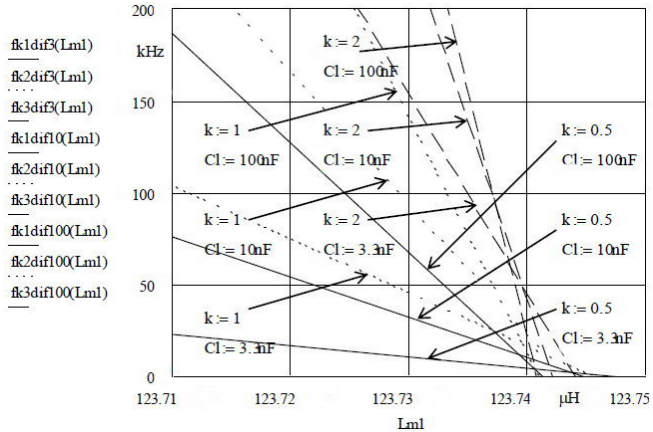
Quartz crystal sensitivity for different *k* for *L*_m1_ and different *C*_L_.

Figures 3 illustrates stepwise capacitance value changes *C*_L_ = 3.3 nF, 10 nF and 100 nF at constant compensation factors *k* for inductances *L*_m1_ and *L*_m2_ = 123.75 μH. One can see that the capacitance *C*_L_ can also be used to set the sensitivity (inclination).

**Figure 4.**
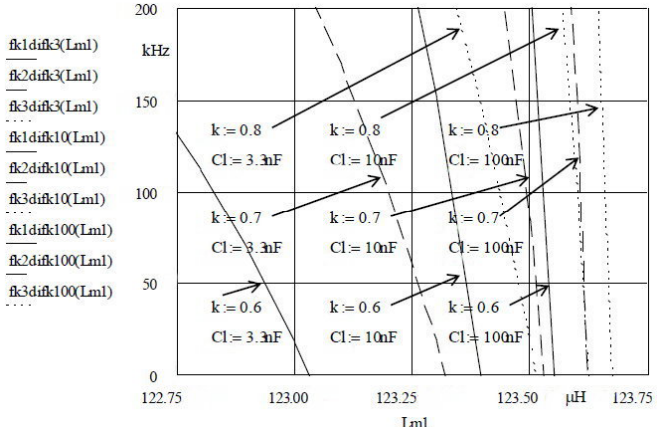
Quartz crystal frequency shift for *k* = 0.6, 0.7, 0.8 for *L*_m1_ and different *C*_L_.

Figure 4 shows that stepwise change of *k* (0.6, 0.7, 0.8) for *L*_m1_ and constant values *k* = 1 for *L*_m2_ = 123.75 μH (for all cases) can change the set oscillator frequency (and to a certain degree the sensitivity as well) by changing the capacitance *C*_L_ = 3.3 nF, 10 nF and 100 nF.

The experimentally selected *k* value combinations show that with the above *C*_L_ values and the compensation ratio *k* for *C*_0_, crystal oscillation frequency (oscillator frequency), sensitivity and linearity with simultaneous compensation of disturbing influences can be set. The pulling sensitivity is the highest at the value *k = 2* (Figure 3) while the stability is lower. At values *k = 0.5* the frequency stability is better, however the pulling sensitivity is lower. The highest sensitivity (∼70 nH/200 kHz) is shown in Figure 4. For the temperature range between 0 °C and 50 °C [14,18], the experimentally measured results of the frequency changes (Programmable Counter/Timer HM 8122) were ±0.00001 Hz (second-to-second stability) and ±0.0001 Hz (minute-to-minute stability) which makes the high-precision measurement of nH changes possible in a given temperature range [14–18,44]. At the highest sensitivity of ∼70 nH/200 kHz at ±0.0001 Hz (minute-to-minute stability), the measurement precision is ∼0.35 fH.

The results illustrated in Figures 2 to 4 provide a comparison of inductance changes *L*_m1_ in the frequency range 0 to 200 kHz. One can observe non-linear changes which occur due to the width of frequency range. The non-linearity of these frequency characteristics depends upon the choice of individual values of the compensation factor *k* and the value of the capacitor *C*_L_.

**Figure 5.**
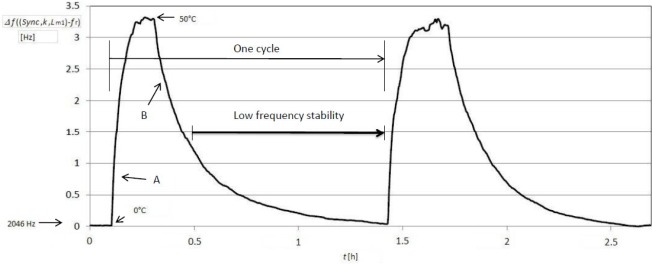
Frequency stability (*f*(*Sync,k,L*_*m*1_) − *f_r_*) occurring when changing the temperature in the range 0–50 °C (measurement time 2.5 h—two cycles).

Figure 5 shows the frequency stability (*f*(*Sync,k,L*_*m*1_) − *f_r_*) (at the beginning of temperature cycling the stable frequency was 2,064 Hz at 0 °C) occurring when changing the temperature in the range between 0 °C and 50 °C at the state *Sync* and fixed value *L*_m1_ = 123.65 μH (Figure 4). The crystal used in the experiment was AT-cut (cut angle: 0′) [37] quartz crystal with temperature change ±5 ppm in the range 10 °C–30 °C. The A and B areas illustrate dynamic change of frequency at the temperature change ranging from 0 °C to 50 °C and back to 0 °C.

**Figure 6.**
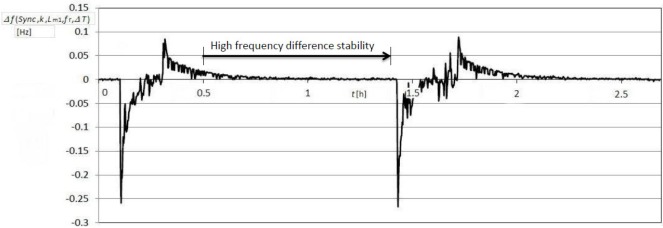
Frequency difference stability $\left(f(\mathrm{Sync},\;k,\;L_{m1}+\Delta L_{m1})-f_{r})-(f(\bar{\mathrm{Sync}},\;k,\;L_{m2})-f_{r}\right)$ at the change of temperature in the range 0 °C–50 °C (measurement time 2.5 h—two cycles).

Figure 6 illustrates frequency stability Δ*f*(*L*_*m*1_) (Equation (8)) during the change of temperature in the range 0 °C–50 °C at the fixed value *L*_m1_ = 123.65 μH (Figure 4) once both frequencies are deducted $\left(f(\mathrm{Sync},\;k,\;L_{m1}+\Delta L_{m1})-f_{r})-(f(\bar{\mathrm{Sync}},\;k,\;L_{m2})-f_{r}\right)$. Deduction of both frequencies in relation to Sync signal is performed by LabVIEW software. In addition, this Figure also illustrates the temperature compensation of the quartz crystal natural temperature characteristics.

The comparison of results in Figures 5 and 6 points to dynamic frequency stability change (Figure 6) and to the high stability (high frequency difference stability— ±0.01 Hz) area when the temperature changes less quickly.

## 7. Conclusions

This article discusses the results of the research aimed at the reduction of the temperature influence of the AT-cut crystal frequency change using the sensor switching method for the inductance change measurement in the nH range. These results show that the use of the dual oscillator switching method excellently compensates quartz crystal non-linear frequency-temperature characteristics, its ageing and oscillator circuit elements. For this purpose AT-cut crystals were used. The value of inductance Δ*L*_m1_ is set by its length and it is produced as a strip line etched on Al_2_O_3_. Basic inductance values *L*_m1_ and *L*_m2_ are two identical elements. The article provides possible oscillator frequency, sensitivity and linearity settings with the compensation factor *k*, the frequency setting (working point) with the capacitance value *C*_L_, the temperature compensation of the crystal characteristics and those of other elements as well as frequency stability after temperature compensation. Also taken into account have been past sensitivity and linearity studies (listed in the references). The great advantage of this method is that it resolves the issue of the sensitivity, linearity and crystal oscillation working point setting as well as the problem of crystal natural temperature characteristics compensation at the same time. The results shown in the article relate to a significantly wider frequency range (0–200 kHz) than is usually covered by practical measurements. With the help of an additional reference frequency and inductance, which makes the differential measurement of inductance changes possible, the output frequency errors can be greatly reduced due to the fact that the frequency counter measures in the range of a few kHz with greater accuracy (more decimal points). The frequency difference in relation to *Sync* signal and the temperature crystal compensation is calculated with the help of the *Sync* signal and the LabVIEW software (Figure 6). This makes this switching method a very interesting tool for the measurement of inductance and small inductance changes which is highly demanded in the sensor industry. The mode of compensation used for the AT-cut crystals opens possibilities for its application in other crystal cuts.

The factors affecting frequency stability such as wide operating temperature range, the use of various types of crystals and drive level should also be considered because a stable oscillator circuit is of vital importance. Stability of the electronic circuit depends upon the circuit type and quality of its elements (elements of the same values must be of the same producer and of the same quality). It is also important that the drive level of the quartz crystal does not exceed 20 μW [15,19,20,32]. These results clearly show that the dual switching method for high-precision small inductance change measurements opens up new possibilities for such measurements compensating at the same time temperature characteristic and other disturbing influences. This makes this new method highly promising for nH range measurements in various fields of physics, chemistry, mechanics, biosensor technology, atomic force, *etc*.

## Figures and Tables

**Table 1.** Quartz data [32].

***f*_0_ (MHz)**	***R* (Ohm)**	***L* (mH)**	***C* (fF)**	***C*_o_ (pF)**	***Q*_q_**	***L*_m1_ (μH)**	***L*_m2_ (μH)**
4.999	10	41	25	8	127,800	122.75–123.75	123.75
